# A novel prenylflavonoid with efficacy in cancer therapy by targeting ceramide-orchestrated signaling

**DOI:** 10.3389/fphar.2026.1862457

**Published:** 2026-06-09

**Authors:** Ruichen Du, Cui Zhang, Hehua Lei, Xin Gao, Qingwei Xiang, Zhiwen Zhang, Yongping Dai, Xinzhi Li, Limin Zhang, Gang Chen

**Affiliations:** 1 State Key Laboratory of Magnetic Resonance and Atomic and Molecular Physics, National Centre for Magnetic Resonance in Wuhan, Innovation Academy for Precision Measurement Science and Technology, CAS, Wuhan, China; 2 University of Chinese Academy of Sciences, Beijing, China; 3 Hubei Shizhen Laboratory, Department of Geriatrics, Hubei University of Chinese Medicine, Hubei Provincial Hospital of Traditional Chinese Medicine, Wuhan, China; 4 The First Affiliated Hospital of Ningbo University, Ningbo, China; 5 School of Pharmacy, Faculty of Medicine, Laboratory for Drug Discovery from Natural Resource, State Key Laboratory of Quality Research in Chinese Medicine, Macau University of Science and Technology, Macao, China

**Keywords:** cancer therapy, cell apoptosis, ceramide, icariside I, metabolomics

## Abstract

**Background:**

Ceramide, acting as an important second messenger, plays a pivotal role in the induction of apoptosis in cancer cells. As one of the active prenylflavonoid ingredients in *Epimedium*, icariside I (GH01) has been shown to exhibit significant anticancer activity. However, the mechanism by which GH01 induces tumor cell apoptosis by targeting ceramide-orchestrated signaling remains unclear.

**Purpose:**

This study aimed to analyze the potential mechanism of GH01 for cancer therapy by generating ceramide formation *in vivo* and *in vitro*.

**Methods:**

A B16F10 melanoma-bearing mouse model was first established and treated with GH01 at different doses. Targeted quantification of ceramide was performed *in vivo* and *in vitro* using HPLC–QQQ-MS. A series of biological assays coupled with immunofluorescence staining and confocal microscope were utilized to analyze the apoptotic mechanism of tumor cells.

**Results:**

GH01 treatment markedly enhanced ceramide levels through the activation of ceramide *de novo* synthesis and sphingomyelin hydrolysis pathways coupled with the simultaneous suppression of sphingosine 1-phosphate generation. Consequently, high levels of ceramide promoted tumor cell apoptosis via both intrinsic and extrinsic apoptosis pathways. Notably, ceramide induced by GH01 interacted with mitochondria, thus releasing cytochrome c, which in turn activated caspase-9 and caspase-3, ultimately triggering cellular apoptosis. Additionally, the supplementation of inhibitors of caspase-3 and exogenous ceramide further confirmed the above apoptosis mechanism.

## 1 Introduction

Melanoma is the most aggressive type of skin cancer triggered by multiple factors such as ultraviolet radiation and environmental toxicant exposure (Arnold et al., 2022; Lawrence et al., 2013). Clinically, melanoma has been treated by surgery, radiotherapy, and chemotherapy with a short window period and high rate of recurrence. The current chemotherapeutic agents widely used for melanoma are cisplatin and dacarbazine (DTIC), two pro-apoptotic drugs with FDA approval. Cisplatin facilitates its therapeutic efficacy by inducing irreversible DNA damage and triggering mitochondrial apoptotic pathways in melanoma cells. However, it can interfere with the DNA of normal proliferating cells, thus inducing certain systemic toxicities (Jordan and Carmo-Fonseca, 2000). As an alkylating antineoplastic agent, DTIC also causes a spectrum of adverse effects such as potential fertility issues, cytotoxicity for normal cells, and drug resistance. BRAF/MEK inhibitor combination therapy, which acts by blocking the MAPK signaling pathway, has been established as the standard of care for BRAF-mutant melanoma, with approximately 60% of melanoma patients harboring key mutations in this cascade. Compared with CTLA-4 blockade, PD-1/PD-L1-inhibitor therapy has demonstrated superior 5-year overall survival (OS), progression-free survival (PFS), and objective response rate (Champion et al., 2025). However, both the therapeutic modalities are associated with the development of drug resistance and exhibit marked heterogeneity in efficacy across distinct melanoma subtypes. Recent studies have revealed that cancer cells can acquire resistance to immunotherapy by coordinately modulating the expression of multiple apoptosis-related genes through genomic copy-number alterations (Wu et al., 2025). Despite the progress in contemporary therapeutic strategies that have led to improved 5-year survival rates for patients with advanced melanoma, outcomes are still marred by limited response rates, treatment resistance, and the occurrence of drug-related adverse events (Luke et al., 2017; Zheng et al., 2025). Therefore, the discovery of novel strategies such as natural molecules with minimal side effects is an urgent task to treat melanoma.

Increasing evidence has shown that the quintessential anti-tumor action of chemotherapeutic drugs is the induction of tumor cellular apoptosis, which is triggered by the mitochondria-mediated intrinsic pathway and death receptor-mediated extrinsic pathway (Savitskaya and Onishchenko, 2015). Within the mitochondria-mediated intrinsic pathway, activation of c-Jun N-terminal kinase (JNK), a member of the MAP kinase superfamily, by some messenger molecules such as ceramide induces disruption of the dynamic equilibrium between cellular survival and apoptosis (Dhanasekaran and Reddy, 2008; Singh et al., 2019). An imbalance between the pro-apoptotic and pro-survival members of the BCL-2 family subsequently leads to an increase of mitochondrial membrane permeability and the release of some proteins such as cytochrome c (Cyt C), which in turn activates caspase-9 and caspase-3, ultimately triggering cellular apoptosis. Additionally, the extrinsic apoptotic pathway is driven by death receptors (e.g., FAS and TRAIL) located on ceramide-enriched membrane platforms (Stancevic and Kolesnick, 2010), which are followed by caspase-8 activation. Hence, evasion of apoptosis is pivotal to the uncontrolled proliferation of cancer cells and tumorigenesis, and the inhibition of cellular apoptosis is recognized as a hallmark of cancer (Hanahan, 2022). Strategies that promote cellular apoptosis are of great interest in the design of anti-cancer drugs.

As an important second messenger signaling molecule, ceramide plays a pivotal role in the induction of apoptosis in cancer cells, and ceramide-based therapies have been shown to provide opportunities for designing new approaches to treat cancer (Bizzozero et al., 2014; Morad and Cabot, 2013; Ogretmen, 2018). It is a core constituent of sphingolipid metabolism and consists of sphinganine coupled with fatty acid chains of diverse carbon lengths (Mullen et al., 2012). These lipids have wide-ranging effects on signal transduction and the regulation of cell function owing to their structural roles in biomembranes. One of the chief functions of ceramide is the potentiation of signaling cascades that lead to cell death and tumor suppression (Morad and Cabot, 2013). *De novo* biosynthesis of ceramide originates from the transformation of serine and palmitoyl-CoA into 3-ketodihydrosphingosine, catalyzed by serine palmitoyltransferase in the endoplasmic reticulum, which subsequently yields dihydroceramide and ceramide (Levy and Futerman, 2010). It has been shown that C16 ceramide encoded by ceramide synthases (CerS5 and CerS6) is a typical molecule implicated in the induction of apoptosis in various cancer cells (Schiffmann et al., 2010; Fekry et al., 2016). Upon exposure to external stressors such as radiation or chemotherapy, sphingomyelin can be transformed into ceramide through sphingomyelin hydrolysis catalyzed by sphingomyelinase (SMase) on the cytoplasmic membrane. The ensuing ceramide acts as a secondary messenger and invokes the JNK pathway, thus initiating apoptosis via the intrinsic mitochondrial pathway (Sanchez et al., 2007; Kolesnick and Fuks, 2003). However, cancer cells have their survival strategies to counteract ceramide-induced apoptosis and exhibit drug resistance by converting ceramide into glucosylceramide via the salvage pathway (Liu et al., 2013; Kitatani et al., 2008). Therefore, successful regulation of ceramide metabolism is the key method for the efficacy of ceramide-based therapies to treat cancer.

Recently, we found that icariside I (GH01, PubChem CID 5745470), one of the active prenylflavonoid ingredients in *Epimedium* (Tan et al., 2016), exhibited significant anticancer activity through the modulation of the gut microbiota and host immunity (Chen et al., 2022; Chen et al., 2021). In this study, we aimed to analyze the mechanisms by which GH01 induces tumor cell apoptosis via ceramide generation *in vitro* and *in vivo*. These findings revealed that GH01 has great potential for tumor chemotherapy through a ceramide-based approach with the induction of cellular apoptosis.

## 2 Materials and methods

### 2.1 Reagents

Hydrochloric acid (HCl), sodium chloride, chloroform, heparin sodium, diethyl ether, and isoflurane anesthesia were obtained from Sinopharm Chemical Co., Ltd (Shanghai, China). Standards of ceramide and sphingomyelins metabolites were purchased from Avanti Polar Lipids (Alabaster, AL, USA). Formic acid (FA), acetonitrile (MeCN), and methanol (MeOH) were purchased from Sigma-Aldrich (Shanghai, China). Icariside I (HPLC >98%) was obtained from Yuanye (CAS: 56725-99-6) Co., Ltd (Shanghai, China) and validated by Guangdong Golden Health Biotechnology Co., Ltd (Guangdong, China) with HPLC described previously (Chen et al., 2022).

### 2.2 *In vitro* experiments

Multiple cell lines including murine melanoma B16F10, human liver cells (L02), human lung cancer cells (A549), human liver cancer cells (HepG2), human breast cancer cells (MCF-7), and human bronchial epithelial cells (16HBE) were obtained from the Chinese Academy of Sciences Cell Bank in Shanghai. These cell lines were cultured in RPMI 1640 or DMEM medium (Gibco, USA) enriched with 10% fetal bovine serum (Cell-box) and 1% penicillin/streptomycin (Gibco, USA) under conditions of 37 °C and a 5% CO_2_ atmosphere. These cells were cultured in T75 flasks (Fisher, USA). Upon exposure to GH01 at different doses, the ceramide content in both B16F10 and L02 cells was analyzed. Both cells were also subjected to pre-treatment with caspase-8-inhibitor Z-IETD-FMK (MCE, USA) and caspase-9-inhibitor Z-LEHD-FMK (MCE, USA) with and without GH01 administration. The function of ceramide in apoptosis was corroborated by introducing exogenous ceramide (C16:0) (Avanti, USA) to the melanoma cell culture. GH01 was solubilized in DMSO, providing a final concentration at 0.5%. The groups of B16F10 melanoma cells treated with GH01 were designated as control (0 μM), low (5 μM), and high (20 μM), with the latter representing an effective concentration for apoptosis induction within a sub-cytotoxic range. An apoptosis inhibitor was administered for 2 h before a 24-h exposure to GH01 (Wang et al., 2019; Zhu et al., 2019; Qiao et al., 2006). The control dose (0 μM), low dose (10 μM), and high dose (40 μM) of ceramide (C16:0) were dissolved in the dodecane/ethanol mixture (2:98, v/v) and introduced into an FBS-free medium for 24 h (Garandeau et al., 2019; Renert et al., 2009). Cells were detached via trypsinization and collected.

### 2.3 Cell viability assay

The cell proliferation and cytotoxicity analyses were conducted using the Cell Counting Kit-8 (CCK-8, Beyotime, China), a methodology leveraging WST-8’s susceptibility to reduction by specific mitochondrial dehydrogenases, producing an orange–yellow formazan. B16F10 cells, post enumeration, were cultured in 96-well plates (Fisher, USA) until reaching a cell density of 60%, following which they were treated with diverse concentrations of GH01 (0, 1, 5, 10, 25, 40, 50, and 100 μM) over a 24-h time-frame. Subsequently, each well was provided with 10 μL of the CCK-8 reagent and incubated at 37 °C for 1 h. The absorbance, in conformance with the manufacturer’s guidelines for CCK-8, was gauged at a wavelength of 450 nm utilizing a microplate reader. This methodology was repeated to confirm CCK-8 measurements for other cell lines, including A549, MCF-7, HepG2, 16HBE, and L02. Cell viability data were normalized to the vehicle control (100%). The final results were presented as the half maximal inhibitory concentration (IC_50_) with 95% confidence intervals (95% CI). Data were fitted using a four-parameter logistic model [log(inhibitor) vs. response-variable slope] in GraphPad Prism, with the top plateau constrained to 100% and the bottom plateau constrained to 0%, to determine the absolute IC_50_.

### 2.4 *In vivo* experiments

Animal experiments received ethical approval from the Animal Ethics Committee at the Innovation Academy of Precision Measurement Science and Technology (APM, no. APM20004A, China). Forty female BALB/c mice aged 6 weeks were obtained from Charles River Co., Ltd (Beijing, China). After acclimatization for 1 week in a specific pathogen-free (SPF) animal house, the mice were randomly allocated into five groups (n = 8) as follows: a control group (normal), a tumor-bearing group (tumor), and three GH01-treated groups at low (5 mg/kg body weight), medium (20 mg/kg body weight), and high doses (80 mg/kg body weight). B16F10 tumor cells were inoculated subcutaneously into the right flank of 24 mice. The tumor volume was measured daily with a Vernier caliper, and the tumor volume was calculated as (length × width × width)/2. When the tumor volume approached approximately 50 mm^3^, the tumor-bearing mice were administered GH01 daily via gavage with a corn oil solution for 1 week, while the control group received an equivalent volume of only corn oil. Tumor dimensions were measured every day. Mice were euthanized via cervical dislocation after 8-h fasting when the tumor volume reached 1,500 mm^3^. The tumor tissues and blood samples were collected and stored at −80 °C for future analyses.

### 2.5 Fluorescence detection

Terminal deoxynucleotidyl transferase dUTP nick end labeling (TUNEL) assays were performed as per the colorimetric TUNEL apoptosis assay kit’s protocols (Beyotime, China). In brief, cells were cultured on slides (BS-14-RC, Biosharp, China) and subjected to GH01 treatment, or not, over 24 h, prior to fixation with 4% paraformaldehyde for half an hour. Subsequently, a 0.3% Triton X-100-induced permeabilization was undertaken, followed by a 30-min incubation period in room-temperature and a subsequent 1-h incubation period at 37 °C in darkness within a moist atmosphere using a pre-formulated TdT-labeled reaction buffer. The slides, post-DAPI (Beyotime, China) staining, were duly sealed. The resultant fluorescent signals were detected via the FITC (525 nm) and DAPI (488 nm) channels. The Reactive Oxygen Species Assay Kit (Beyotime, Shanghai, China), based on the DCFH-DA method, was utilized to analyze changes in endogenous ROS levels. DCFH-DA, a non-fluorescent compound, transforms into highly fluorescent DCF upon ROS generation within cells. Cells cultured on slides were treated with and without GH01 for 24 h and subsequently stained in serum-free medium with 10 μM DCFH-DA oxide dye for 20 min. The excitation and emission wavelengths were recorded at 488 nm and 525 nm, respectively. The fluorescent probe JC-1, recognized for its extensive use in detecting mitochondrial membrane potential, was utilized in this study. In cells undergoing apoptosis and demonstrating low mitochondrial membrane potential, JC-1 is unable to accumulate in the mitochondrial matrix and, consequently, fluoresces green as monomers. However, in non-apoptotic cells exhibiting higher mitochondrial membrane potential, JC-1 aggregates in the mitochondrial matrix and emits red fluorescence. After cell culture and treatment with and without GH01 for 24 h, the JC-1 staining solution was introduced and incubated at 37 °C for 20 min. The excitation wavelengths for JC-1 monomer and JC-1 aggregates were noted to be 525 nm and 588 nm, respectively. For immunofluorescence experiments, B16F10 cells were cultured on cell slides fixed using 4% paraformaldehyde after GH01 treatment. Then, the membrane was permeabilized with 0.2% Triton X-100 dissolved in PBS. The cell slides were washed in PBS, post-blocked with 2% bovine serum albumin (BSA), and incubated for 1 h at 37 °C followed by incubation with primary antibodies, caspase-3 (Proteintech, USA), overnight at 4 °C. After two washes, the slides were subsequently incubated with secondary goat anti-rabbit antibody for 1 h at 37 °C and stained with DAPI. All the above experimental procedures were performed strictly according to the manufacturer’s instructions, and all cell staining was observed under a Nikon fluorescence microscope. Images were analyzed using ImageJ software.

### 2.6 Quantification of ceramide metabolites

Quantitative evaluation of ceramide metabolites in melanoma cells and in both plasma and tumor tissues of mice with and without tumor was carried out using an ultra-high-performance liquid chromatography system (Agilent 1290) in conjunction with a 6460 triple quadrupole mass spectrometer (UHPLC–QQQ-MS, Agilent Technologies, Inc.) operating in multiple reaction monitoring (MRM) modes. The extraction methodology utilized was built upon previously established protocols (Kasumov et al., 2010; Bielawski et al., 2009; Zhang et al., 2022). Further details about the procedures of ceramide measurements are provided in the Supplementary Material, along with the parameters of ceramide detection by LC–MS (Supplementary Table S1).

### 2.7 Quantitative real-time PCR (QPCR)

Total RNA from melanoma cells and hepatocytes was isolated utilizing the RNAiso Plus reagent (TaKaRa). Reverse transcription into cDNA was undertaken with the assistance of a quantitative polymerase chain reaction (QPCR) reagent kit (One-Step gDNA removal, TransGen Biotech). Real-time PCR was conducted using Green qPCR SuperMix (TransGen Biotech) in an ABI StepOne real-time PCR system (Applied Biosystems). QPCR conditions were stipulated as 40 cycles at 95 °C for 30 s, followed by 95 °C for 3 s, and then 60 °C for 30 s. The melting curve was used to determine the specificity of the product. All the reactions were normalized with the expression levels of glyceraldehyde-3-phosphate dehydrogenase (Gapdh) (Supplementary Table S2), and the expression levels were calculated using the 2^−ΔΔCT^ method.

### 2.8 Statistical analysis

The data were statistically analyzed using GraphPad 7.0 software. All values were analyzed by one-way ANOVA and two-tailed Student’s t-test and presented as the mean ± standard deviation. Significant differences were marked as *p < 0.05, **p < 0.01, and ***p < 0.001.

## 3 Results

### 3.1 Effect of GH01 on cell proliferation

**FIGURE 1:**
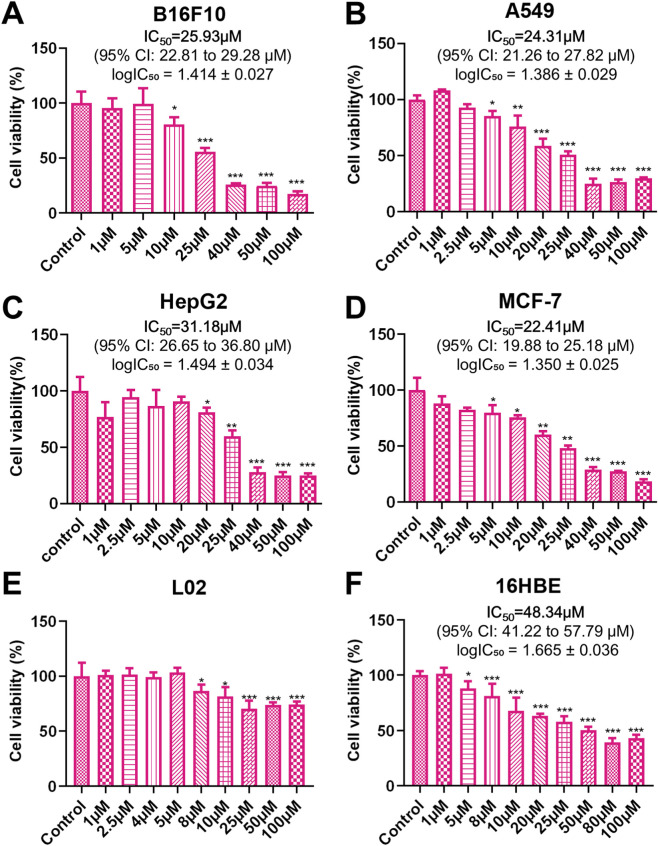
GH01 affects cell proliferation of **(A)** B16F10, **(B)** A549, **(C)** HepG2, **(D)** MCF-7, **(E)** L02, and **(F)** 16HBE cells. Values are presented as the means ± SD (n = 4). Absolute IC_50_ values and 95% confidence intervals (95% CI) were derived by non-linear regression using a four-parameter logistic model with the top constrained to 100% and the bottom to 0%. The IC_50_ (95% CI) and logIC_50_ ± SE values are as follows: B16F10, 25.93 μM (22.81 μM–29.28 μM), logIC_50_ = 1.414 ± 0.027; A549, 24.31 μM (21.26 μM–27.82 μM), logIC_50_ = 1.386 ± 0.029; HepG2, 31.18 μM (26.65 μM–36.80 μM), logIC_50_ = 1.494 ± 0.034; MCF-7, 22.41 μM (19.88 μM–25.18 μM), logIC_50_ = 1.350 ± 0.025; L02 and 16HBE, ≥48.34 μM. Statistical significance is denoted as *p < 0.05, **p < 0.01, and ***p < 0.001 compared to the control group. *P*-values were obtained by one-way ANOVA with multiple comparisons.

A range of cancer cell types including melanoma cells (B16F10), lung cancer cells (A549), liver cancer cells (HepG2), and breast cancer cells (MCF-7) coupled with human liver cells (L02) and human bronchial epithelial cells (16HBE) were treated with GH01 at different doses (from 1 to 100 μM) for 24 h, and the viability of these cells was assayed by CCK-8 (Figure 1). In comparison with cancer cells (IC_50_ values ranging from 22.41 to 31.18 μM; Figures 1A–D), both normal cell lines (L02 and 16HBE) exposed to GH01 showed markedly higher IC_50_ values (>48.34 μM) (Figures 1E,F), indicating a favorable selectivity profile against cancer cells (Figures 1E,F). These results implied that GH01 has potential anticancer effects on various cancer cells, while it exerts a relatively minimal effect on the proliferation of normal cells.

### 3.2 GH01 inhibits tumor growth and promotes apoptosis

**FIGURE 2:**
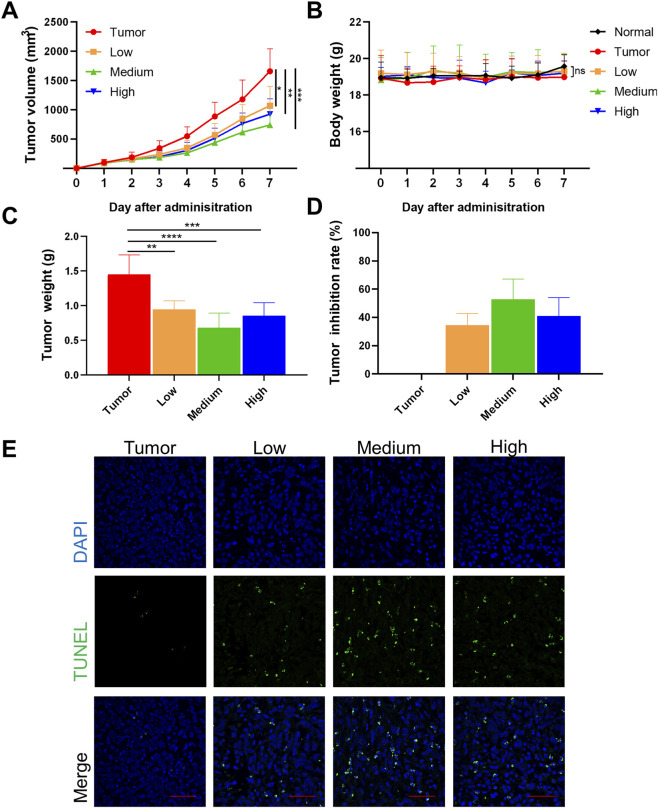
GH01 inhibits tumor growth via promoting cell apoptosis. **(A)** Tumor volume, **(B)** body weight, **(C)** tumor weight, and **(D)** tumor inhibition rate in melanoma-bearing mice. **(E)** Representative image of TUNEL staining for detection of apoptosis in tumor tissues of each group (green, TUNEL; blue, DAPI/nuclei; scale bar = 50 μm; n = 3). The values are presented as the means ± standard deviation (n = 6). Statistical significance is denoted as *p < 0.05, **p < 0.01, and ***p < 0.001 compared to the tumor group. *P*-values were obtained by one-way ANOVA with multiple comparisons or two-way ANOVA.

GH01 low (5 mg/kg body weight)-, medium (20 mg/kg body weight)-, and high (80 mg/kg body weight)-dose treatment induced significant reduction in both the tumor volume and weight of B16F10 tumor-bearing mice without significant changes in body weight (Figures 2A–D). Specifically, oral administration of GH01 at a dosage of 20 mg/kg body weight led to a tumor inhibition rate of 52.84%, which is higher than that (34.61%) of mice treated with a relatively lower dosage (5 mg/kg body weight). Moreover, histological examination of tumor tissue sections showed that GH01 markedly induced apoptosis in tumor cells (Figure 2E). These phenotypic data indicated that GH01 inhibits tumor growth via the induction of tumor cells apoptosis.

### 3.3 GH01 upregulates ceramide levels in tumor tissue and tumor cells

**FIGURE 3:**
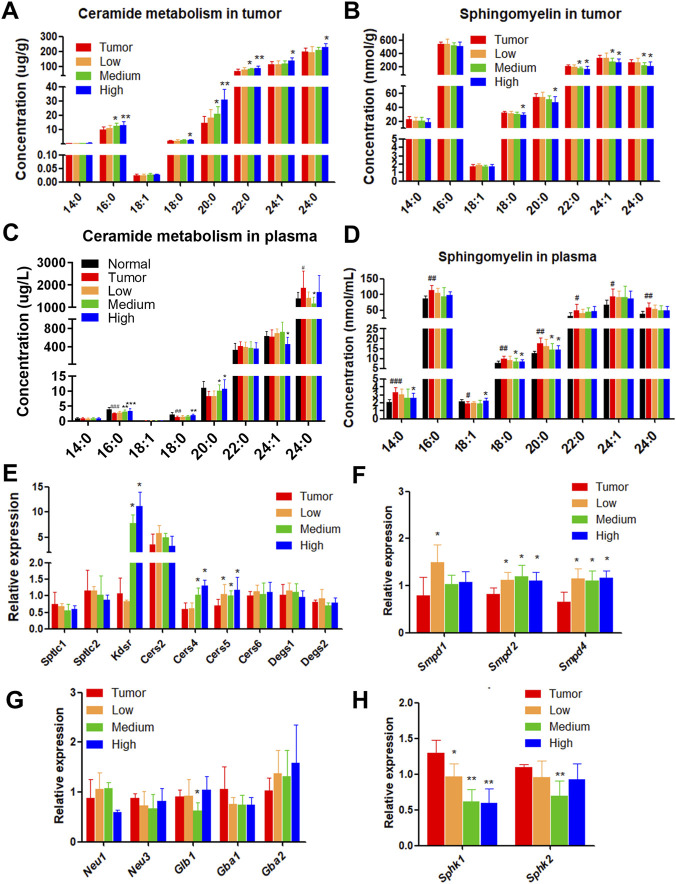
GH01 upregulates ceramide levels in plasma and tumor tissues of mice. Quantification of ceramides and sphingomyelins in tumor tissues **(A,B)**. Quantification of ceramides and sphingomyelins in plasma **(C,D)**. QPCR analysis of genes involved in *de novo* ceramide synthesis **(E)**, sphingolipid hydrolysis **(F)**, salvage synthesis pathways **(G)**, and sphingosine kinase **(H)** in tumor tissues. Values are presented as the means ± standard deviation (n = 6). Statistical significance is denoted as ^#^ *p* < 0.05, ^##^ *p* < 0.01, and ^###^ *p* < 0.001 compared to the normal group; *p < 0.05, **p < 0.01, and ***p < 0.001 compared to the tumor group. *P-*values were obtained by one-way ANOVA with multiple comparisons.

In order to analyze the mechanisms of apoptosis of tumor cells induced by GH01, we next performed quantification of ceramide *in vivo* and *in vitro* (Supplementary Figure S1). Targeted metabolomics analyses showed that GH01 treatment dose-dependently upregulated the levels of ceramides, including 16:0, 18:0, 20:0, 22:0, 24:0, and 24:1 in tumor tissue (Figure 3A) and 16:0, 18:0, and 20:0 in plasma of tumor-bearing mice (Figure 3C). Concomitantly, significant decreases in the levels of sphingomyelin were observed in both tumor tissue (18:0, 20:0, 22:0, 24:0, and 24:1) (Figure 3B) and plasma (14:0, 18:0, and 20:0) (Figure 3D). Based on these changes of ceramide concentrations, we further examined how treatment with GH01 affected the transcriptional regulation of genes encoding key enzymes involved in the ceramide synthesis pathways. Ceramide *de novo* synthesis-related mRNAs such as 3-ketodihydrosphingosine reductase (Kdsr) and ceramide synthases (CerS4 and CerS5) were significantly upregulated in tumor tissues by GH01 treatment (Figure 3E). GH01 administration also markedly upregulated the levels of mRNAs such as sphingomyelin phosphodiesterase (Smpd1, Smpd2, and Smpd4) involved in the sphingomyelin hydrolysis pathway producing ceramide (Figure 3F). However, the levels of ceramide synthesis-related mRNAs, including sphingomyelin phosphodiesterase 1 (Neu1 and Neu3) and glucosylceramidase (Gba1 and Gba2), involved in the salvage pathway, were not significantly changed in tumor tissues of mice by GH01 treatment (Figure 3G). In addition, GH01 treatment markedly downregulated the mRNAs levels of sphingosine kinases (Sphk1 and Sphk2) (Figure 3H), encoding key enzymes for the conversion of ceramide to sphingosine-1-phosphate (S1P), which is a bioactive lipid associated with tumor cell survival, proliferation, angiogenesis, chemotherapy resistance, and cancer cell invasion (Pyne et al., 2018).

**FIGURE 4:**
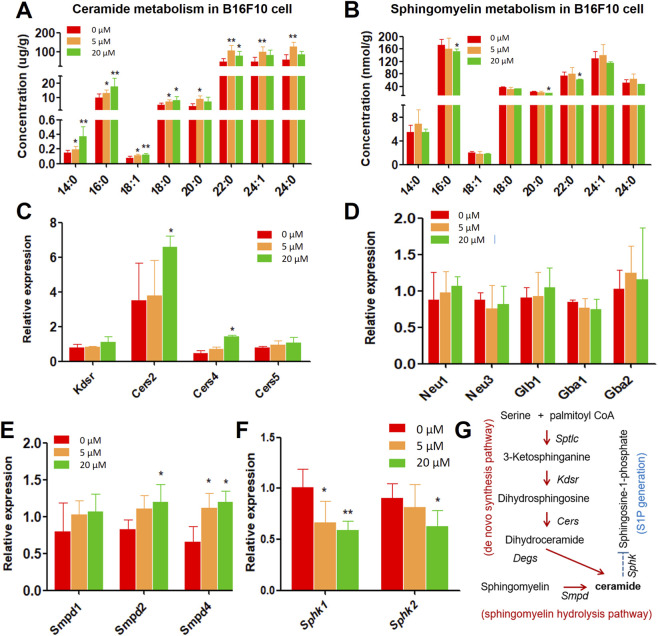
GH01 upregulates ceramide levels in B16F10 cells. Quantification of ceramides **(A)** and sphingomyelins **(B)** in melanoma cells. **(C)** QPCR analysis of genes involved in *de novo* ceramide synthesis, **(D)** ceramide salvage synthesis, **(E)** sphingolipid hydrolysis pathway, and **(F)** sphingosine kinase in tumor cells. **(G)** Schematic illustration of the ceramide metabolic pathway. Values are presented as the means ± standard deviation (n = 6). Statistical significance is denoted as *p < 0.05, **p < 0.01, and ***p < 0.001 compared to the control group. *P-*values were obtained by one-way ANOVA with multiple comparisons.

Similarly, treatment with low (5 μM) and high (20 μM) doses of GH01 for 24 h also markedly elevated the levels of ceramides (14:0, 16:0, 18:0, 18:1, 20:0, 22:0, 24:0, and 24:1) (Figure 4A), accompanied by significant decreases in the levels of sphingomyelin (16:0, 20:0, and 22:0) (Figure 4B) in B16F10 cells. Ceramide synthesis-related mRNAs such as CerS2, CerS4, Smpd2, and Smpd4 were significantly upregulated by GH01 in B16F10 cells (Figures 4C,E), while no significant changes in the mRNA levels involved in the salvage pathway were observed *in vitro* (Figure 4D). GH01 treatment also markedly downregulated the mRNA levels of sphingosine kinases (Sphk1 and Sphk2) (Figure 4F). Interestingly, GH01 administration displayed a negligible impact on ceramide metabolism in the liver and plasma of healthy mice (Supplementary Figures S2A,B) and normal L02 cells (Supplementary Figure S3A). Consistently, the ceramide synthesis-related mRNAs involved in ceramide *de novo* synthesis, sphingomyelin hydrolysis, and salvage pathways together with sphingosine kinases were not significantly altered by GH01 treatment (Supplementary Figures S3B-F). These results indicate that GH01 promotes ceramide synthesis but inhibits S1P generation both in tumor-bearing mice and tumor cells (Figure 4G), while having very little impact on healthy mice and normal cells.

### 3.4 GH01 induces tumor cell apoptosis

**FIGURE 5:**
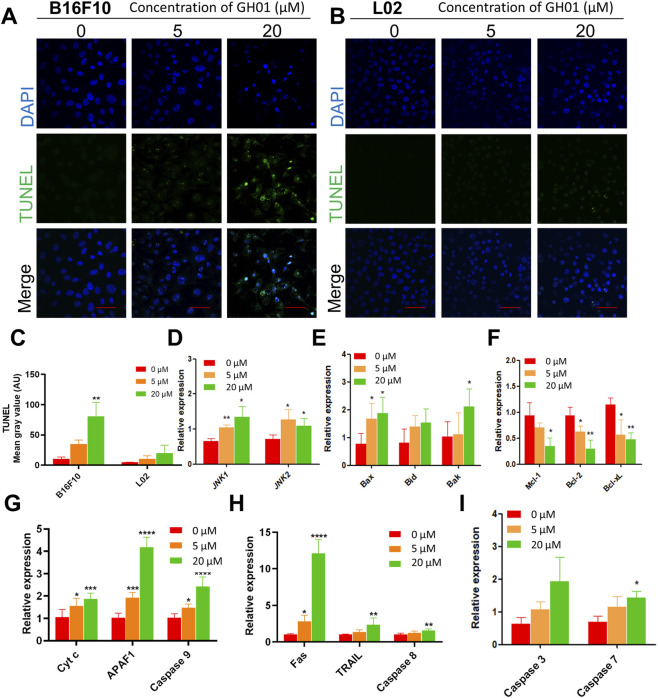
GH01 induces apoptosis in B16F10 cells. Representative TUNEL staining images showing apoptosis in B16F10 **(A)** and L02 **(B)** cells (green, TUNEL; blue, DAPI/nuclei; scale bar = 50 μm). **(C)** Quantification of TUNEL fluorescence (n = 3). **(D)** QPCR analysis of *JNK*, pro-apoptotic protein **(E)**, anti-apoptotic protein **(F)**, mitochondrial apoptotic pathway **(G)**, death receptor pathway **(H)**, and apoptotic protein **(I)** in tumor cells. Values are presented as the means ± standard deviation (n = 6). Statistical significance is considered as *p < 0.05, **p < 0.01, and ***p < 0.001 compared to the control group. *P-*values were obtained by one-way ANOVA with multiple comparisons.

Given that GH01 treatment markedly elevated the ceramide levels in tumor cells, we next analyzed the mechanisms of ceramide that trigger tumor cell death. Here, TUNEL staining revealed that GH01 treatment at a high dose (20 μM) caused a remarkable increase in the apoptotic activity of the B16F10 cell cohort, while it exhibited no significant impact on the apoptotic activity of the L02 cell cohort (Figures 5A–C). Within the intrinsic apoptotic pathway, ceramide accumulation was found to stimulate the c-Jun N-terminal kinase (JNK) pathway, which is manifested by significant upregulation in the mRNA levels of JNK1 and JNK2 (Figure 5D), thus leading to the disturbance of the dynamic equilibrium among the BCL-2 protein family members that regulate apoptosis and cell survival. This led to enhanced mRNA levels of pro-apoptotic genes (Bax and Bak) (Figure 5E) and the simultaneous repression of anti-apoptotic genes (Mcl-1, Bcl-2, and Bcl-xL) (Figure 5F). Subsequently, apoptosis-related proteins such as cytochrome C (Cyt C), released from the mitochondrial electron transport chain, apoptotic peptidase activating factor 1 (Apaf1), and caspase-9 were strongly induced by GH01 treatment in tumor cells, thus triggering mitochondrial-mediated intrinsic apoptosis (Figure 5G). Regarding the extrinsic apoptotic pathway, significant upregulation in the expression of Fas and TNF-related apoptosis-inducing ligand (TRAIL) receptor was noticeable in tumor cells after GH01 treatment. Concurrent enhancement and activation of the mRNA level of caspase-8 indicated activation of the extrinsic apoptotic pathway (Figure 5H). Ultimately, caspase-3 and caspase-7 were activated, indicating apoptosis of B16F10 cells by both intrinsic and extrinsic apoptotic pathways (Figure 5I). Remarkably, GH01 treatment did not provoke the corresponding apoptotic pathways in L02 cells, as shown by no significant changes in the mRNA levels of the corresponding genes (Supplementary Figure S4). Collectively, these observations indicate that GH01 induces tumor cellular apoptosis by elevating ceramide levels via initiating the apoptotic signaling pathways.

### 3.5 GH01 induces ceramide–mitochondria interaction

**FIGURE 6:**
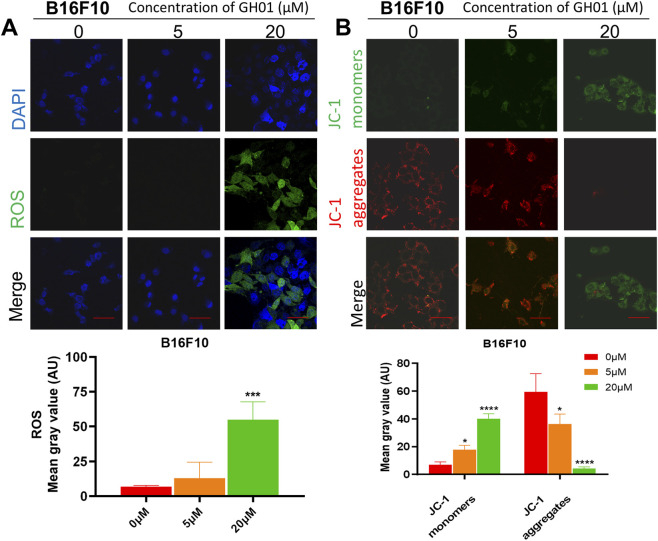
GH01 induces ROS generation and changes in mitochondrial membrane permeability. **(A)** Representative TUNEL staining images indicating apoptosis in B16F10 cells (green, TUNEL; blue, DAPI/nuclei; scale bar = 50 μm) and the quantification of TUNEL fluorescence. **(B)** Representative JC-1 staining images indicating membrane potential in B16F10 cells (green, JC-1 monomers; red, JC-1 aggregates; scale bar = 50 μm) and the quantification of JC-1 staining fluorescence. Values are presented as the means ± standard deviation (n = 3). Statistical significance is considered as *p < 0.05, **p < 0.01, and ***p < 0.001 compared to the control group. *P-*values were obtained by one-way ANOVA with multiple comparisons.

**FIGURE 7:**
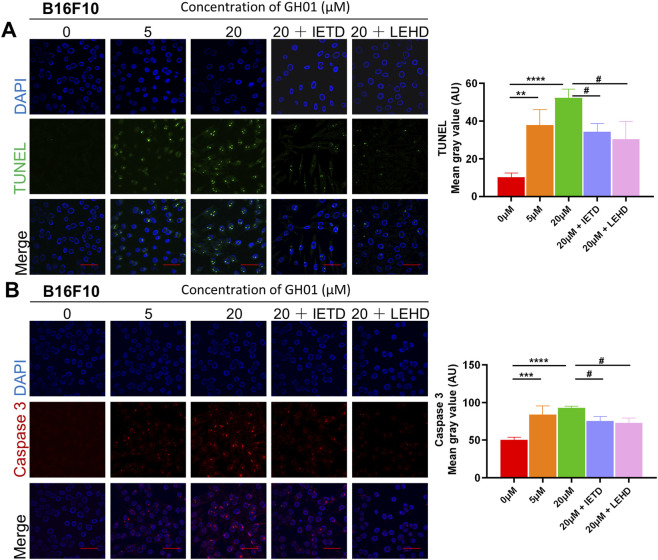
GH01 induces apoptosis in B16F10 cells via death receptor and mitochondrial pathways. B16F10 cells were treated with GH01 along with the caspase-8 inhibitor (Z-IETD-FMK, IETD) or the caspase-9 inhibitor (Z-LEHD-FMK, LEHD). **(A)** Representative TUNEL staining images indicating apoptosis in B16F10 cells (green, TUNEL; blue, DAPI/nuclear; scale bars = 50 μm). Quantification of TUNEL fluorescence. Values are presented as the means ± SD (n = 3). **(B)** Representative immunofluorescence images of B16F10 cells with caspase-3 expression (red, caspase-3; blue, DAPI/nuclear; scale bar = 50 μm). Quantification of caspase-3 fluorescence. Values are presented as the means ± SD (n = 3). Statistical significance was determined as *p < 0.05, **p < 0.01, and ***p < 0.001 compared to the control group; #*p* < 0.05, ##*p* < 0.01, and ###*p* < 0.001 compared to the 20-μM group. *P*-values were obtained by one-way ANOVA with multiple comparisons.

Reactive oxygen species (ROS) measurements showed that GH01 treatment induced marked generation of ROS in tumor cells (Figure 6A). Furthermore, mitochondrial membrane potential collapsed in tumor cells upon GH01 treatment, which was manifested by the upregulation of JC-1 monomers and downregulation of JC-1 aggregates with GH01 treatment at different doses (Figure 6B). Consequently, markedly elevated permeability of the mitochondrial membrane led to the efflux of some proteins such as Cyt C from the mitochondria, thereby activating the intrinsic apoptotic pathway of tumor cells. We next used caspase-8 inhibitor (Z-IETD-FMK, IETD) and caspase-9 inhibitor (Z-LEHD-FMK, LEHD) to verify the induction of apoptotic pathways in tumor cells by GH01. Notably, the results of TUNEL staining showed that GH01 initiated tumor cell apoptosis, while the treatments with the addition of inhibitors of caspase-8 and caspase-9 partially mitigated tumor apoptosis (Figure 7A). Immunofluorescence detection and quantitative analysis of the caspase-3 protein level demonstrated that GH01 treatment markedly upregulated the protein level of caspase-3, which was partially downregulated with the introduction of inhibitors (Figure 7B). Taken together, these data indicate that GH01 induces ceramide–mitochondria interaction, thereby causing tumor cell apoptosis via activating the death receptor pathway.

### 3.6 Ceramide (16:0) directly facilitates tumor cell apoptosis

**FIGURE 8:**
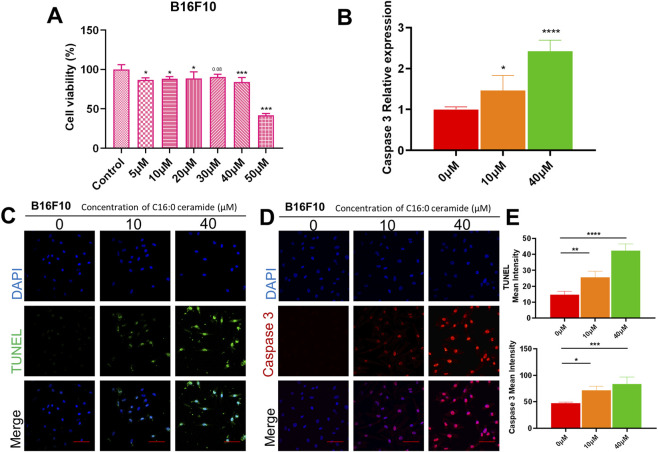
Exogenous ceramide (16:0) induces apoptosis in B16F10 cells. Impact of exogenous ceramide on cell proliferation **(A)** and expression of apoptotic proteins **(B)** in B16F10 cells. **(C)** Representative TUNEL staining images illustrating apoptosis in B16F10 cells (green, TUNEL; blue, DAPI/nuclear; scale bar = 50 μm). **(D)** Representative immunofluorescence images of B16F10 cells with caspase-3 expression (red, caspase-3; blue, DAPI/nuclear; scale bar = 50 μm). **(E)** Quantification of TUNEL and caspase-3 fluorescence. Values are presented as the means ± SD (n = 3). Statistical significance was determined as *p < 0.05, **p < 0.01, and ***p < 0.001 compared to the control group. *P-*values were obtained by one-way ANOVA with multiple comparisons.

The elevated level of endogenous C-16-ceramide is known as a common feature of the triggers for apoptosis induction (Blaess et al., 2015). To analyze whether ceramide directly or indirectly triggers tumor cell apoptosis, different doses (0, 5, 10, 20, 30, 40, and 50 μM) of exogenous ceramide (16:0) were used to treat B16F10 cells. B16F10 cells were treated with low (10 μM) and high (40 μM) doses of ceramide (16:0) for 24 h. Cell viability measurements showed that ceramide-exposure significantly inhibited tumor cell growth in a dose-dependent manner (Figure 8A). Moreover, TUNEL assays indicated that C16 ceramide dose-dependently induced tumor cell apoptosis (Figures 8C,E) accompanied by elevation in caspase-3 at both the mRNA (Figure 8B) and protein levels (Figures 8D,E). These results indicate that ceramide can directly facilitate tumor cell apoptosis and ceramide formation can be induced by exposure to GH01 chemotherapy through promoting ceramide synthesis.

## 4 Discussion

During the past decades, the incidence of melanoma has continued to rise in the world, and melanoma is often refractory to commonly used anticancer drugs (Mattia et al., 2018). At present, several therapeutic choices against melanoma have been approved for clinical use, including conventional chemotherapy, molecularly targeted therapy, and immunotherapy (Guo et al., 2021; Flaherty et al., 2012). However, it is difficult to obtain promising results using these options for metastatic melanoma due to its high heterogeneity and high genetic mutational rate (Klemen et al., 2020). Numerous studies have shown that natural flavonoids have medicinal applications for cancer prevention and treatment (Chen et al., 2016; Ding et al., 2022). More recently, we discovered and demonstrated that GH01 is a novel non-toxic prenylflavonoid in *Epimedium*, known as a traditional herbal medicine in China, and it exhibits strong anti-cancer activity by modulating the gut microbiota and host systemic immunity (Chen et al., 2022; Chen et al., 2021). As a bioactive flavonoid, GH01 has great potential for cancer immunotherapy by blocking the kynurenine–AhR pathway and tumor immune escape. In addition, we also found that GH01 is able to effectively ameliorate osteoporosis and protect against inflammation (Wu et al., 2023). In the current study, we further analyzed the new mechanisms of cancer chemotherapy through which GH01 treatment induced ceramide formation *in vivo* and *in vitro* via the promotion of *de novo* and sphingomyelinase (SMase) pathways, thus causing tumor cell apoptosis.

Relatively lower IC_50_ values of various cancer cells than normal cells after treatment with GH01 indicates that GH01 can effectively inhibit tumor cell proliferation with minimal impacts on the proliferation of normal cells, which were further confirmed by our *in vivo* experiments that GH01 exhibited a marked amelioration in the tumor phenotype of melanoma-bearing mice without significant effects on the organs of normal mice. Mechanistically, GH01 induced tumor cell apoptosis through the activation of both intrinsic and extrinsic apoptosis pathways. Actually, a variety of chemotherapeutic drugs such as DTIC, etoposide, and daunorubicin trigger these two apoptotic signaling pathways to provoke tumor cell apoptosis and death (Day et al., 2009; Oh et al., 2012). Of particular note in this study was the marked activation of the intrinsic apoptosis pathway (also regarded as the mitochondrial pathway) by GH01-boosted accumulation of ceramide. Ceramide is a key component of sphingolipids that serve structural roles in biomembranes and signal regulation of cell function as an important second messenger molecule (Summers et al., 2019; Castro et al., 2014). Here, GH01 treatment significantly promoted *de novo* synthesis and sphingomyelin hydrolysis pathways, thus generating ceramide *in vivo* and *in vitro*. Subsequently, ceramide was transferred directly from the cellular membrane to the mitochondria, leading to mitochondrial ceramide accumulation and mitochondrial outer membrane permeabilization (MOMP), which was confirmed by significant decreases in the mitochondrial membrane potential (Colombini, 2017; Chang et al., 2015; Abou-Ghali and Stiban, 2015). Supportive evidence can be found that ceramide induced the activation of JNK and BAX translocation to mitochondria, manifested by significant upregulation in the mRNA levels of JNK1 and JNK2 and pro-apoptotic genes (Bax and Bak) (Chen et al., 2008; Chipuk et al., 2012). Ceramide accumulation promoted the release of Cyt c from the mitochondria into the cytoplasm, thereby leading to the activation of caspase-9 and caspase-3. Simultaneous repression of the mRNA levels of anti-apoptotic genes (Mcl-1, Bcl-2, and Bcl-xL) indicated that enriched ceramide inhibited tumor-cell apoptotic resistance to GH01 chemotherapy, which was further confirmed by suppressing the generation of S1P. S1P and its substrate sphingosine are produced by the hydrolysis of ceramide, and S1P generation is a cell’s anti-apoptotic process. It can be seen that the maintenance of the balance between ceramide and S1P is pivotal for ceramide-based therapeutics of cancer (Kim et al., 2018; Pyne et al., 2018). Together, these findings indicated that ceramide formation triggered by GH01 facilitated tumor cell apoptosis via simultaneously upregulating the mitochondrial pro-apoptotic and inhibiting the anti-apoptotic pathways.

Significant upregulation in the expression of Fas and TNF-related apoptosis-inducing ligand (TRAIL) receptor in tumor cells after GH01 treatment indicated that the extrinsic apoptosis pathway was also initiated by GH01, since Fas and TRAIL are the cell membrane proteins known as death receptors. Ceramide-enriched cellular membrane platforms facilitate the transmission of Fas and TRAIL signals and the formation of death-inducing signaling complexes (DISCs), thus concurrently recruiting adapter proteins such as caspase-8 (Grassmé et al., 2003). Previous studies showed that the activation of initiator caspase-8 led to the cleavage of activator proteins such as BH3-interacting domain death agonist (BID), which is subsequently translocated into mitochondria, linking the extrinsic and intrinsic pathways, thereby leading to the release of Cyt c (Flores-Romero et al., 2022). Here, exogenous C16 ceramide exposure directly induced tumor cell apoptosis, indicating that ceramide is a tumor suppressor and that ceramide metabolism is the key to the efficacy of ceramide-based therapies for cancer. We note that JNK and caspase activation was inferred from transcriptional upregulation and pharmacological inhibition, and direct measurement of p-JNK/JNK and cleaved caspases will be required to further validate this pathway. However, the addition of inhibitors of caspase-8 and 9 treatments partially mitigated tumor apoptosis, which further validated that GH01-triggered ceramide formation promoted tumor cell apoptosis via both the intrinsic and extrinsic apoptotic pathways. Based on the apoptotic signaling pathways, several flavonoid compounds such as scutellarin (SCU) and icariside II have been discovered to induce Fas-mediated extrinsic cell apoptosis in Hep3B cells and the death receptor apoptosis pathway in human breast cancer cells (MCF-7), respectively (Sang Eun et al., 2019; Wu et al., 2018).

In summary, this study revealed that GH01, a novel prenylflavonoid in the traditional herb *Epimedium*, exhibits strong anti-cancer potential by targeting ceramide-orchestrated signaling *in vivo* and *in vitro*. Specifically, GH01 effectively triggers *de novo* synthesis and sphingomyelin hydrolysis pathways of ceramide, thus causing ceramide accumulation and tumor cell apoptosis via the activation of apoptotic signaling pathways. Interestingly, GH01 displays negligible impacts on normal cells and healthy mice. These findings provide preclinical evidence that GH01, through the modulation of ceramide metabolism, might be a promising candidate for further anticancer evaluation.

## Data Availability

The original contributions presented in the study are included in the article/Supplementary Material, further inquiries can be directed to the corresponding authors.
