# Supplementary material for: A novel prenylflavonoid with efficacy in cancer therapy by targeting ceramide-orchestrated signaling
Source: Front Pharmacol. 2026 Jun 9;17:1862457. doi: 10.3389/fphar.2026.1862457 (PMC13287425; doi:10.3389/fphar.2026.1862457)
Supplement: Supplementary file 1 [file Supplementaryfile1.docx]

Supplemental material

# Material and Methods

## Quantification of ceramide metabolites

Ceramide and sphingomyelin were analyzed by ultra-high-performance liquid chromatography (Agilent 1290) and triple quadrupole mass spectrometer (Agilent 6460) (UHPLC-QQQ-MS) with internal standards of (17:0-ceramide and sphingomyelin, Sigma). Samples were separated at 45°C using ZORBAX Eclipse plus C8 column (2.1 m × 150 mm, 1.8 μm Agilent). The mobile phases A and B were composed of ultra-pure water and methanol containing 2.5 mM ammonium acetate. For ceramide analysis, the phase B gradient was 94% to 98% at 0.35 mL/min for 3 min, and then 98% B for 9 min at 0.25 mL/min. The chromatographic efferent were introduced into QQQ-MS for positive mode multi-reaction monitoring (MRM). Mother and child ions and characteristic daughter ions were selected specifically in a single injection. MS/MS conversions for individual standards are described in the Supplementary Materials section. sphingomyelin standard calibration and samples were analyzed using the UPLC-MS system. The UPLC conditions of sphingomyelin were 2.0 mM NH_4_OCOH aqueous solution and 1.0 mM NH_4_OCOH in MeOH containing 0.2% HCOOH as solvent A and B, and the flow rate was 0.35 mL/min. The quantification of ceramides and Sph was based on the individual standard curve established with area_-analyte_/area _internal std_. The MS/MS transitions (m/z) for C14 was (492.4 → 264), C16 was (520.4 → 264), C17 was (534.4 → 264), C18:1 was (546.4 → 264), C18 was (548 → 264), C20 was (576.5 → 264), C22 was (604.4 → 264), C24:1 was (630.4 → 264), and C24 was (632.4 → 264).

**Table S1.** Parameters of ceramides detection method based on LC-MS

| Ceramides | Linear curve | | Limit of quantification（nmol/L） | Limit of detection（nmol/L） | Linear range  (nmol/L) |
| --- | --- | --- | --- | --- | --- |
|  | fitting formula | R^2^ |  |  |  |
| d18:1/2:0 | y=5330.81 x+ 40148.92 | 0.995 | 0.29 | 0.10 | 0.2～146 412.88 |
| d18:1/4:0 | y=6175.95 x+51158.40 | 0.995 | 0.54 | 0.18 | 0.5～135 318.00 |
| d18:1/6:0 | y=8237.30 x+67770.45 | 0.995 | 1.26 | 0.42 | 1.2～125 786.16 |
| d18:1/8:0 | y=8556.62 x+52529.33 | 0.996 | 0.47 | 0.16 | 0.4～117 508.81 |
| d18:1/10:0 | y=8027.10 x+57107.72 | 0.994 | 0.44 | 0.15 | 0.4～110 253.58 |
| d18:1/12:0 | y=8006.94 x+52597.28 | 0.997 | 0.42 | 0.14 | 0.4～103 842.16 |
| d18:1/14:0 | y=8418.31 x+41557.01 | 0.996 | 0.39 | 0.13 | 0.39～98 135.43 |
| d18:1/16:0 | y=7681.01 x+38502.09 | 0.997 | 0.37 | 0.12 | 0.37～93 023.26 |
| d18:1/17:0 | y=6606.31 x+41304.01 | 0.996 | 0.36 | 0.12 | 0.36～90 661.83 |
| d18:1/18:1 | y=4562.08 x+141862.17 | 0.998 | 0.35 | 0.12 | 0.35～17 683.47 |
| d18:1/18:0 | y=5665.98 x+38660.01 | 0.995 | 0.18 | 0.06 | 0.18～17746.23 |
| d18:1/20:0 | y=5784.24 x+45374.00 | 0.994 | 0.17 | 0.06 | 0.17～16 849.20 |
| d18:1/22:0 | y=4137.20 x+35607.86 | 0.992 | 0.32 | 0.11 | 0.32～16 090.10 |
| d18:1/24:1 | y=4186.11 x+32724.18 | 0.994 | 0.31 | 0.10 | 0.31～15 396.46 |
| d18:1/24:0 | y=3964.02 x+34505.30 | 0.992 | 0.77 | 0.26 | 0.77～15 444.02 |

| Ceramides | Recovery rate / % | | | Matrix effect /% | | |
| --- | --- | --- | --- | --- | --- | --- |
|  | low | medium | high | low | medium | high |
| d18:1/2:0 | 97.78 | 122.19 | 81.66 | 113.73 | 94.30 | 85.02 |
| d18:1/4:0 | 107.58 | 99.41 | 92.17 | 89.88 | 80.65 | 81.97 |
| d18:1/6:0 | 112.47 | 81.90 | 80.55 | 109.68 | 88.47 | 105.68 |
| d18:1/8:0 | 74.89 | 82.27 | 86.90 | 91.59 | 107.71 | 92.78 |
| d18:1/10:0 | 86.21 | 85.49 | 118.48 | 92.20 | 82.92 | 116.87 |
| d18:1/12:0 | 105.27 | 80.96 | 116.82 | 92.00 | 84.28 | 106.32 |
| d18:1/14:0 | 103.14 | 93.11 | 91.99 | 90.54 | 106.80 | 111.50 |
| d18:1/16:0 | 104.75 | 114.67 | 122.39 | 83.07 | 84.90 | 71.31 |
| d18:1/18:1 | 73.02 | 111.27 | 99.36 | 94.00 | 88.58 | 82.16 |
| d18:1/18:0 | 92.25 | 93.70 | 94.21 | 81.21 | 106.28 | 102.06 |
| d18:1/20:0 | 111.30 | 87.30 | 79.92 | 94.55 | 102.54 | 116.70 |
| d18:1/22:0 | 100.99 | 83.59 | 83.17 | 76.46 | 98.66 | 82.95 |
| d18:1/24:1 | 113.10 | 78.50 | 85.65 | 79.02 | 83.83 | 84.34 |
| d18:1/24:0 | 102.67 | 86.69 | 80.24 | 122.48 | 96.02 | 103.43 |
| RSD/% | 13.08 | 14.94 | 15.97 | 14.22 | 10.45 | 15.38 |

**Table S2.** Primers used for quantitative real-time PCR.

| Genes | Forward primer (5’ -3’) | Reverse primer (5’-3’) |
| --- | --- | --- |
| *Sptlc1* (Mouse) | CGAGGGTTCTATGGCACATT | GGTGGAGAAGCCATACGAGT |
| *Sptlc2* (Mouse) | TCACCTCCATGAAGTGCATC | CAGGCGTCTCCTGAAATACC |
| *Kdsr* (Mouse) | TCCAGTGGCATTGGGAAGTG | CTTCTCTTGTGCCTGCTTTATGA |
| *CerS1* (Mouse) | CCACCACACACATCTTTCGG | GGAGCAGGTAAGCGCAGTAG |
| *CerS2* (Mouse) | AAGTGGGAAACGGAGTAGCG | ACAGGCAGCCATAGTCGTTC |
| *CerS3* (Mouse) | CAGGCGAGGAGTATCCTGTG | CTCTCCGACCAGAACCATTTTC |
| *CerS4* (Mouse) | GGATTAGCTGATCTCCGCAC | CCAGTATGTCTCCTGCCACA |
| *CerS5* (Mouse) | GTGGAACCCAATGACACCCT | CGTTGGAGGCTTGTCCTGAT |
| *CerS6* (Mouse) | AAGCCAATGGACCACAAACT | TGCTTGGAGAGCCCTTCTAAT |
| *Degs1* (Mouse) | AATGGGTCTACACGGACCAG | TGGTCAGGTTTCATCAAGGAC |
| *Degs2* (Mouse) | AAGCCAATGGACCACAAACT | TGCTTGGAGAGCCCTTCTAAT |
| *Smpd1* (Mouse) | GTTACCAGCTGATGCCCTTC | AGCAGGATCTGTGGAGTTG |
| *Smpd2* (Mouse) | AGCAGGATCTGTGGAGTTG | CTCCAGCCATGAAGCTCAAC |
| *Smpd4* (Mouse) | ACCTGGCCCTCAATCCATTTG | ATAGGCACAGTCCGAAGTACG |
| *Neu1* (Mouse) | GGACCGCTGAGCTATTGGG | CGGGATGCGGAAAGTGTCTA |
| *Neu3* (Mouse) | ATGGAGGCCACATTACCTGG | TCTGGCACCTCTCAGTAACAT |
| *Glb1* (Mouse) | GCACGGCATCTATAATGTCACC | GTATCGGAATGGCTGTCCATC |
| *Gba1* (Mouse) | GCCAGGCTCATCGGATTCTTC | CACGGGGTCAAGAGAGTCAC |
| *Gba2* (Mouse) | GGCTGTGCCGAAAGAGATTC | ATCCTGGGGTCCACTATCCTC |
| *Sphk1* (Mouse) | ACTGATACTCACCGAACGGAA | CCATCACCGGACATGACTGC |
| *Sphk2* (Mouse) | ACAGCGACTACGCCCAAAG | GTGGGTAGGTGTAGATGCAGA |
| *Fas* (Mouse) | CTGCGATTCTCCTGGCTGTGAA | CAACAACCATAGGCGATTTCTGG |
| *TRAIL* (Mouse) | GGAAGACCTCAGAAAGTGGCAG | TTTCCGAGAGGACTCCCAGGAT |
| *JNK 1* (Mouse) | CGCCTTATGTGGTGACTCGCTA | TCCTGGAAAGAGGATTTTGTGGC |
| *JNK 2* (Mouse) | GTCAGTGGGTTGCATCATGGGA | ACTCTGCGGATGGTGTTCCTAG |
| *Bax* (Mouse) | AGGATGCGTCCACCAAGAAGCT | TCCGTGTCCACGTCAGCAATCA |
| *Bid* (Mouse) | CCACAACATTGCCAGACATCTCG | TCACCTCATCAAGGGCTTTGGC |
| *Bak* (Mouse) | GGAATGCCTACGAACTCTTCACC | CAAACCACGCTGGTAGACGTAC |
| *Mcl1* (Mouse) | AGCTTCATCGAACCATTAGCAGAA | CCTTCTAGGTCCTGTACGTGGA |
| *Bcl2* (Mouse) | CCTGTGGATGACTGAGTACCTG | AGCCAGGAGAAATCAAACAGAGG |
| *Bcl-xl* (Mouse) | TTCGGGATGGAGTAAACTGGG | CTCCTTGTCTACGCTTTCCAC |
| *Cyt C* (Mouse) | CCAAATCTCCACGGTCTGTTC | ATCAGGGTATCCTCTCCCCAG |
| *APAF1* (Mouse) | CACGAGTTCGTGGCATATAGGC | GGAAATGGCTGTCGTCCAAGGA |
| *Caspase8* (Mouse) | CAACTTCCTAGACTGCAACCG | TCCAACTCGCTCACTTCTTCT |
| *Caspase9* (Mouse) | GCTGTGTCAAGTTTGCCTACCC | CCAGAATGCCATCCAAGGTCTC |
| *Caspase3* (Mouse) | ATGGAGAACAACAAAACCTCAGT | TTGCTCCCATGTATGGTCTTTAC |
| *Caspase7* (Mouse) | AAGACGGAGTTGACGCCAAG | CCGCAGAGGCATTTCTCTTC |
| *GAPDH* (Mouse) | AAATGGTGAAGGTCGGTGTGAAC | CAACAATCTCCACTTTGCCACTG |
| *β-actin* (Mouse) | AGCCATGTACGTAGCCATCC | CTCTCAGCTGTGGTGGTGAA |
| *Sptlc1* (Human) | GGTGGAGATGGTACAGGCG | TGGTTGCCACTCTTCAATCAG |
| *Sptlc2* (Human) | AACGGGGAAGTACGGAACG | CCCCACATACGTGAGCACAG |
| *Kdsr* (Human) | TGCTGGTAAATTGTGCAGGAA | GCCCAGGTAATTGATGCTCAT |
| *CerS2* (Human) | TTCTGGTGGGAACGTCTGTG | CTTTGGCGTAGACACGTCCAT |
| *CerS4* (Human) | TGGTGCTGCTGTTACACGAT | CCTGTTGCTGATGGACTCGT |
| *CerS5* (Human) | GGTCACCATTGGGCTTATCTCC | GTGTCACAGAGCCGCTGATACT |
| *CerS6* (Human) | GACGCAATCAGGAGAAGCCAAG | GGTAGTTGTACCAGCAATGCCTC |
| *Degs2* (Human) | CCAACATTCCTGGAAAAAGTCTTC | CCAACATTCCTGGAAAAAGTCTTC |
| *Smpd1* (Human) | GCTGGCTCTATGAAGCGATGGC | AGAGCCAGAAGTTCTCACGGGA |
| *Smpd2* (Human) | TGGTGCTCAACGCCTATGTGAC | GTCTGCCTTCTTGGATGTGTGG |
| *Neu1* (Human) | TCCAAGGCTGAGAACGACTTCG | TCAGCAAAGGCGAGAAGAGTGC |
| *Gba* (Human) | TGCTGCTCTCAACATCCTTGCC | TAGGTGCGGATGGAGAAGTCAC |
| *Ugcg* (Human) | CTTGGTTCACGGGCTGCCTTAC | GAAACCAGTTACATTGGCAGAGAT |
| *Sphk1* (Human) | GCTGGCAGCTTCCTTGAACCAT | GTGTGCAGAGACAGCAGGTTCA |
| *Sphk2* (Human) | GAGGAAGCTGTGAAGATGCCTG | GAGCAGTTGAGCAACAGGTCGA |
| *JNK1* (Human) | GACGCCTTATGTAGTGACTCGC | TCCTGGAAAGAGGATTTTGTGGC |
| *JNK2* (Human) | TACGTGGTGACACGGTACTACC | CACAACCTTTCACCAGCTCTCC |
| *Bax* (Human) | TCAGGATGCGTCCACCAAGAAG | TGTGTCCACGGCGGCAATCATC |
| *Bak* (Human) | TTACCGCCATCAGCAGGAACAG | GGAACTCTGAGTCATAGCGTCG |
| *Mcl1* (Human) | CCAAGAAAGCTGCATCGAACCAT | CAGCACATTCCTGATGCCACCT |
| *Bcl2* (Human) | ATCGCCCTGTGGATGACTGAGT | GCCAGGAGAAATCAAACAGAGGC |
| *Caspase3* (Human) | GGAAGCGAATCAATGGACTCTGG | GCATCGACATCTGTACCAGACC |
| *Caspase7* (Human) | CGGAACAGACAAAGATGCCGAG | AGGCGGCATTTGTATGGTCCTC |
| *GAPDH* (Human) | ACAACTTTGGTATCGTGGAAGG | GCCATCACGCCACAGTTTC |
| *β-actin* (Human) | GAGAAAATCTGGCACCACACC | GGATAGCACAGCCTGGATAGCAA |

**Figures**


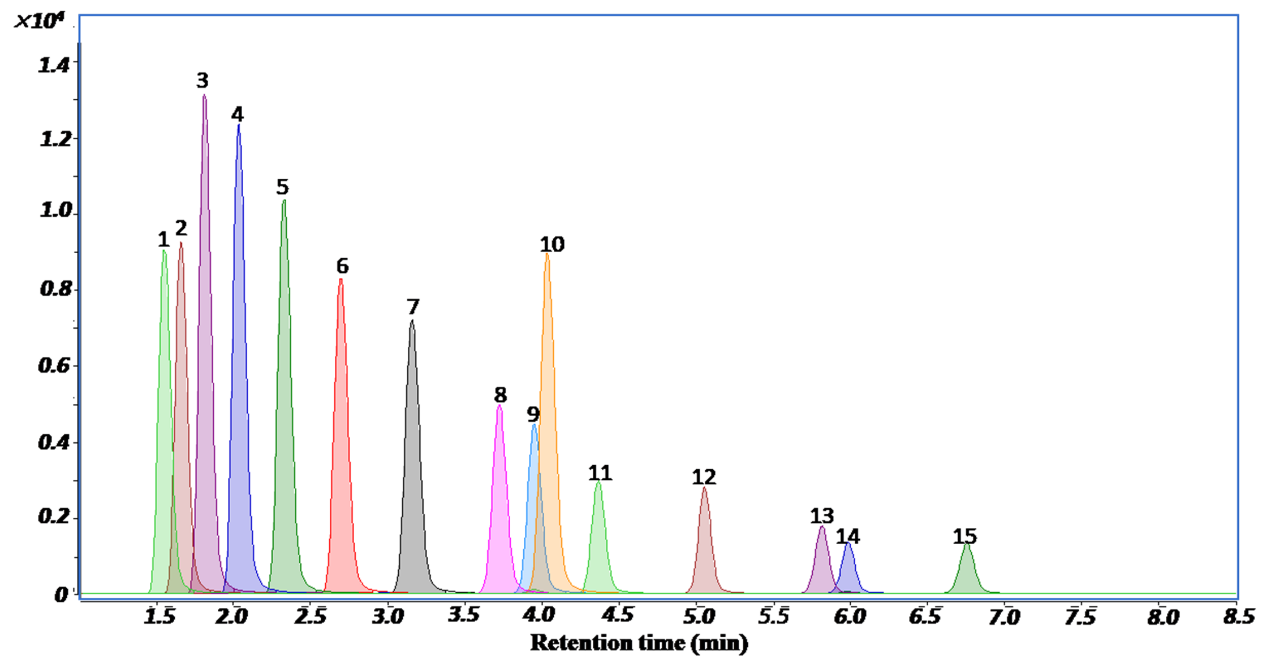


**Figure S1**. The total ion chromatogram of 14 ceramides standards mix, 1-d18:1/2:0, 2-d18:1/4:0, 3-d18:1/6:0, 4-d18:1/8:0, 5-d18:1/10:0, 6-d18:1/12:0, 7-d18:1/14:0, 8-d18:1/16:0, 9-d18:1/18:1, 10-d18:1/17:0 (Internal standard), 11-d18:1/18:0, 12-d18:1/20:0, 13-d18:1/22:0, 14-d18:1/24:1(15z), 15-d18:1/24:0. For an example of ceramide [d18:1/2:0], d18:1 represents the basic skeleton of ceramides, 2:0 means that the chain length is 2 carbon atoms (C2) and the number of unsaturated bonds is 0.

**
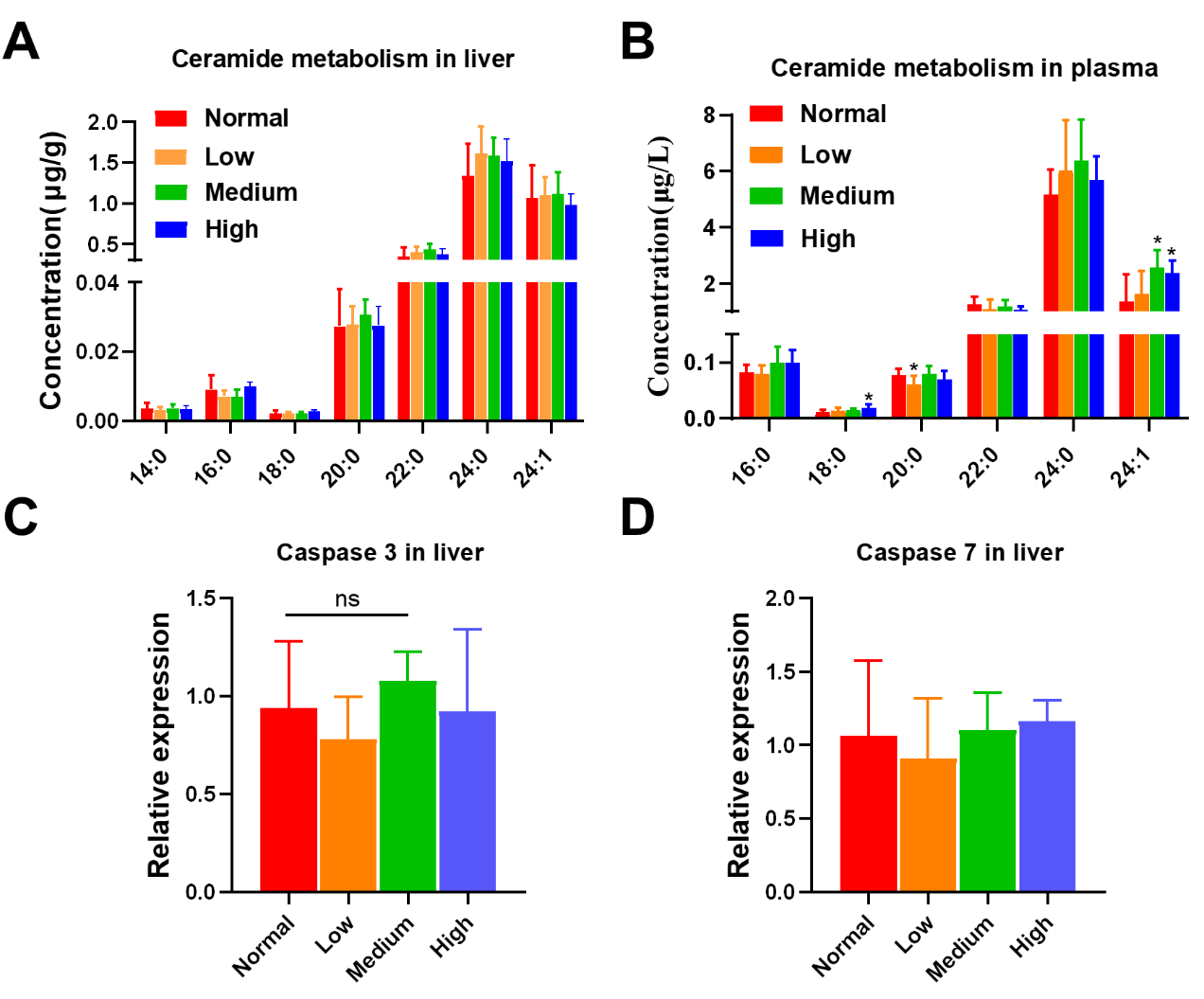
**

**Figure S2**. Effect of GH01 on ceramide levels in healthy mouse. **(A)** Quantitative analysis of ceramide levels in healthy mouse liver and plasma **(B)**. **(C)** RT-PCR analysis of caspase 3 and caspase 7 **(D)** in liver tissues. Values are presented as means ± SD (n = 6). Statistical significance was determined as **p* < 0.05, ***p* < 0.01, and ****p* < 0.001 compared to the normal group.


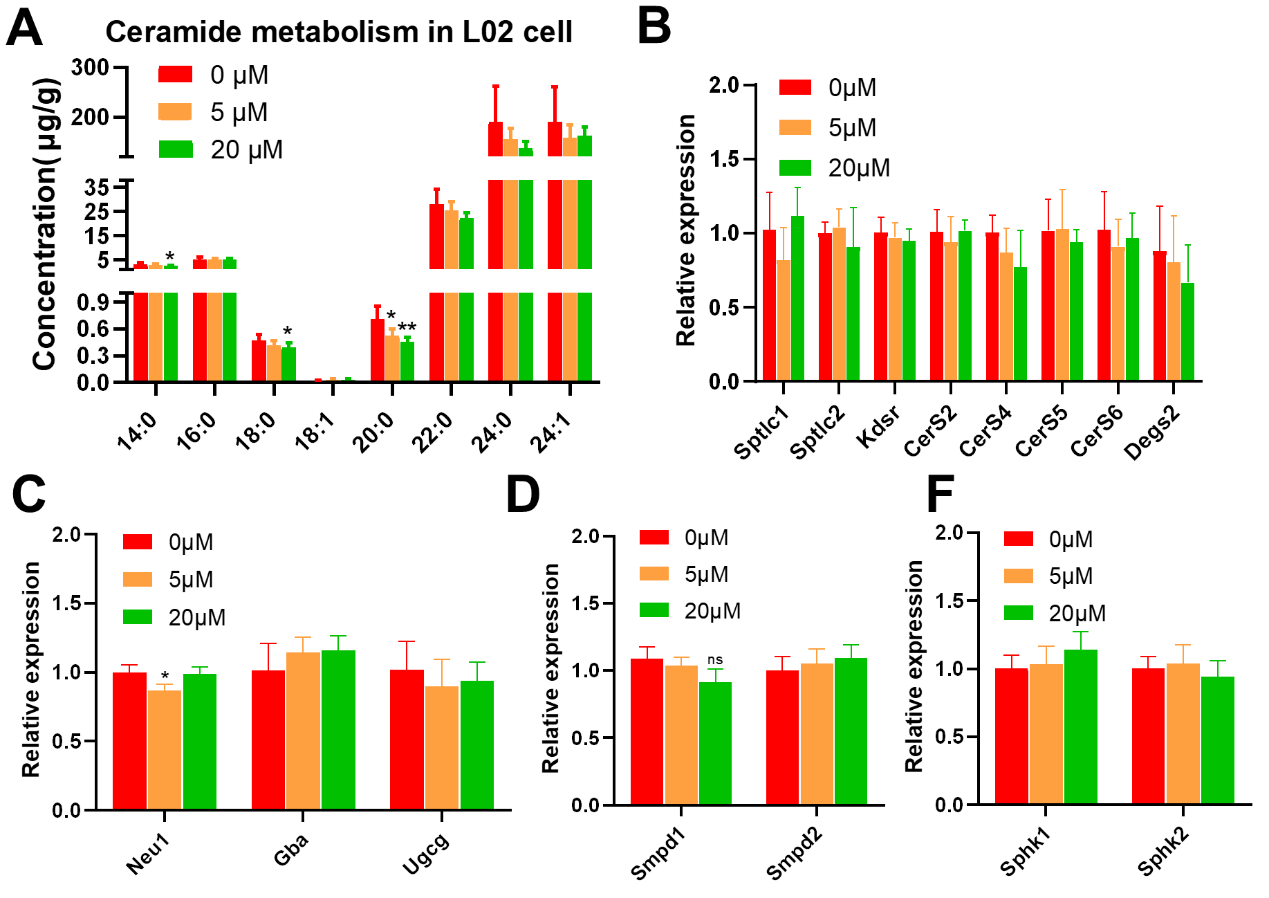


**Figure S3**. Impact of GH01 on ceramide metabolism in L02 cells. **(A)** Quantitative analysis of ceramide levels in L02 cells. **(B)** mRNA levels of genes involved in ceramide de novo synthesis pathway, **(C)** salvage synthesis pathway, **(D)** sphingolipid hydrolysis pathway, and **(E)** sphingosine kinase in L02 cells. Values are presented as means ± SD (n = 6). Statistical significance was determined as **p* < 0.05, ***p* < 0.01, and ****p* < 0.001 compared to the control group.


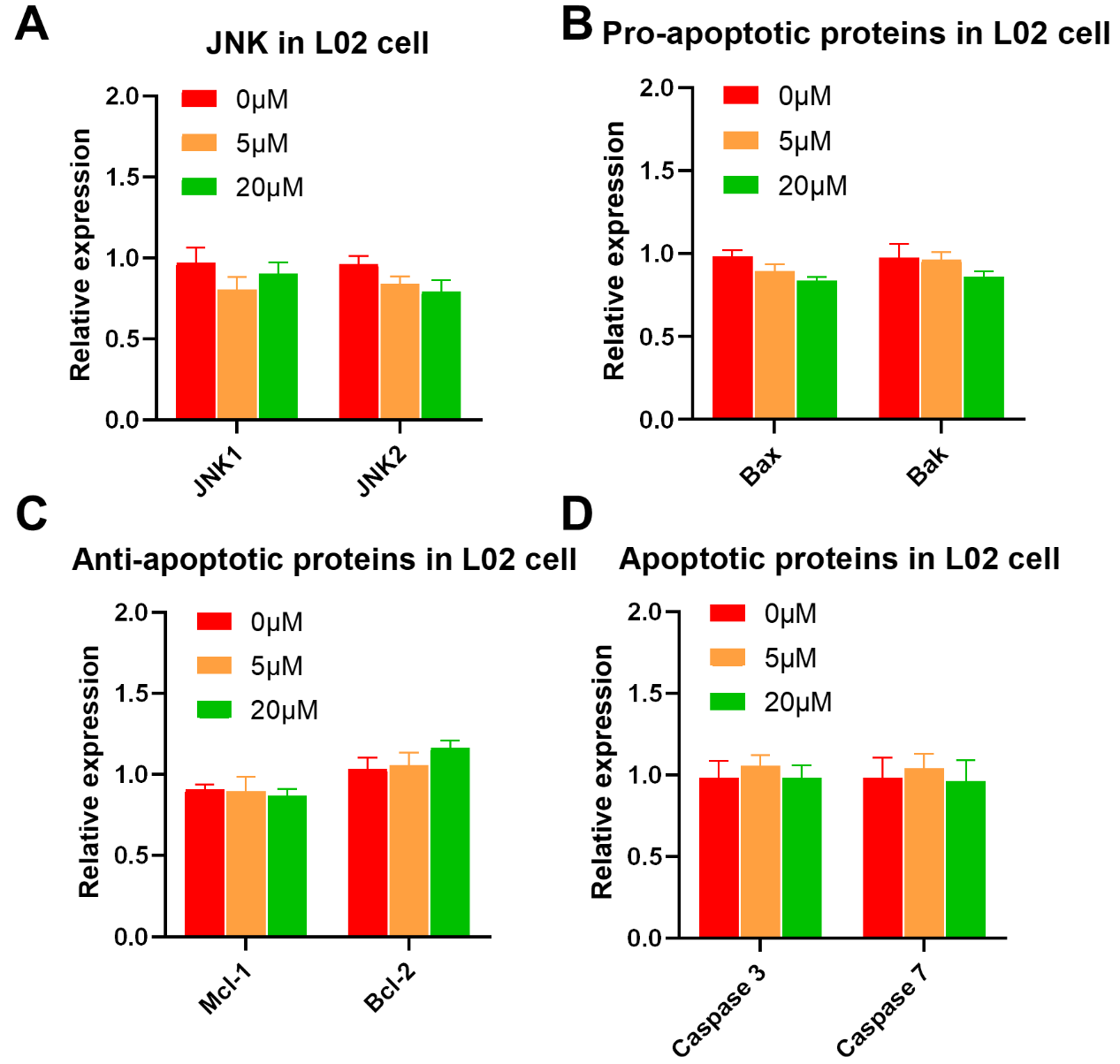


**Figure S4**. Effect of GH01 on apoptosis pathways in L02 cells. **(A)** mRNA levels of *JNK1* and *JNK2* in L02 cells. **(B)** mRNA levels of *Bax* and *Bak* in L02 cells. **(C)** mRNA levels of *Mcl-1* and *Bcl-2* in L02 cells. **(D)** mRNA levels of *Caspase 3* and *Caspase 7* in L02 cells. Value are presented as means ± SD (n = 6). Statistical significance was determined as **p* < 0.05, ***p* < 0.01, and ****p* < 0.001 compared to the control group.
